# Predicting the cover and richness of intertidal macroalgae in remote areas: a case study in the Antarctic Peninsula

**DOI:** 10.1002/ece3.4463

**Published:** 2018-08-19

**Authors:** Jonne Kotta, Nelson Valdivia, Tiit Kutser, Kaire Toming, Merli Rätsep, Helen Orav‐Kotta

**Affiliations:** ^1^ Estonian Marine Institute University of Tartu Tallinn Estonia; ^2^ Instituto de Ciencias Marinas y Limnológicas Facultad de Ciencias Universidad Austral de Chile Valdivia Chile; ^3^ Centro FONDAP de Investigación en Dinámica de Ecosistemas Marinos de Altas Latitudes (IDEAL) Valdivia Chile

**Keywords:** Antarctica, benthic vegetation, biotic patterns, climate change, remote sensing, Sentinel‐2A

## Abstract

Antarctica is an iconic region for scientific explorations as it is remote and a critical component of the global climate system. Recent climate change causes a dramatic retreat of ice in Antarctica with associated impacts to its coastal ecosystem. These anthropogenic impacts have a potential to increase habitat availability for Antarctic intertidal assemblages. Assessing the extent and ecological consequences of these changes requires us to develop accurate biotic baselines and quantitative predictive tools. In this study, we demonstrated that satellite‐based remote sensing, when used jointly with *in situ* ground‐truthing and machine learning algorithms, provides a powerful tool to predict the cover and richness of intertidal macroalgae. The salient finding was that the Sentinel‐based remote sensing described a significant proportion of variability in the cover and richness of Antarctic macroalgae. The highest performing models were for macroalgal richness and the cover of green algae as opposed to the model of brown and red algal cover. When expanding the geographical range of the ground‐truthing, even involving only a few sample points, it becomes possible to potentially map other Antarctic intertidal macroalgal habitats and monitor their dynamics. This is a significant milestone as logistical constraints are an integral part of the Antarctic expeditions. The method has also a potential in other remote coastal areas where extensive *in situ* mapping is not feasible.

## 1 INTRODUCTION

Antarctica is a desolate and largely unexplored region, and at the same time, it is a critical component of the global climate system (Kennicutt et al., 2014). As a consequence of current climate change, warming in Antarctica (Vaughan et al., 2003) has resulted in a dramatic retreat of ice and a rise in sea level, with associated impacts to society and the global system (Shepherd et al., 2012). For instance, climate change‐related mass loss of Antarctic ice sheets is one of the main drivers of global sea‐level rise (DeConto & Pollard, 2016). Such rapid environmental change raises fundamental questions about the capacity of the Antarctic ecosystem to cope with these impacts.

Until recently, Antarctic coastal ecosystems were thought of as virtually devoid of macroscopic life. This barren appearance is related to frequent ice scour that removes a large proportion of macrobenthic intertidal organisms during winter (Pugh & Davenport, 1997). In the past decade, however, a different picture has emerged (Kennicutt et al., 2014). The intertidal algal communities actually develop rapidly during austral summer when large areas of the coast are devoid of ice (Griffiths & Waller, 2016). This cyclic process of recolonization—in combination with substrate heterogeneity and biotic interactions—is thought to underpin a high spatial variability in the structure of intertidal Antarctic macroalgal assemblages (Valdivia et al., 2014). Such communities are restricted to regions with milder abiotic environmental conditions (Griffiths & Waller, 2016). However, rapid warming and increasing risk of species introductions in Antarctica (Chown et al., 2015) can largely modify the current colonization pattern. In addition, glacial retreat has a huge potential to expose ever‐increasing areas of intertidal habitat (Clark, Raymond, Riddle, Stark, & Johnston, 2015) and thereby even further increase the role of intertidal macroalgal habitats in Antarctic ecosystems.

Antarctic macroalgal assemblages have very high rates of endemism (Wiencke & Clayton, 2002), and therefore, any dramatic shift in species distribution ranges, such as related to global climate change, could not only jeopardize the integrity of Antarctic coastal ecosystems, but also lead to irreplaceable loss of continental‐scale biodiversity. To date, intertidal habitats of Antarctica still represent a great unknown, as biological exploration of this continent has been limited to a few bays. Moreover, most scientists visit the continent only for a few summer months each year (e.g., Valdivia, Pardo, Macaya, Huovinen, & Gómez, 2018; Waller, 2013; Zacher, Wulff, Molis, Hanelt, & Wiencke, 2007). Macroalgal assemblages in rocky intertidal areas of the Antarctic Peninsula—comprised of green, brown, and red algae—have been shown to be more diverse than those in southern South America (Griffiths & Waller, 2016). In addition, the intertidal species richness of King George Island is higher than that of any other Antarctic or sub‐Antarctic island (Griffiths & Waller, 2016), but the latter may reflect an uneven sampling effort across these regions. Thus, standardized and comparable data of Antarctic intertidal habitats are of utmost importance for understanding how these systems function and against which to compare current and future trends.

Such quantitative baseline data can be gained using novel remote sensing methods calibrated with *in situ* ground‐truthing techniques. The recently launched Sentinel‐2A satellite equipped with a multispectral imager (i.e., an instrument simultaneously recording about 10 bands across the electromagnetic spectrum) primarily aims at monitoring variability in land surface conditions. Due to its wide observed area and high revisit time (i.e., the time elapsed between observations of the same point on earth by a satellite), it supports monitoring and understanding of global change and their impact on biota. The launch of Sentinel‐2B furthermore reduced the revisit time down to 2–3 days in Antarctica and thereby significantly increased the chance of acquiring cloud‐free imagery in such frequently cloudy areas. Moreover, the satellite provides high‐spatial‐resolution data (10 m) and thereby it is possible to monitor the shifting patterns of small‐scale features not detected by most previous sensors (MERIS, MODIS). We are aware that the spaceborne hyperspectral instruments (i.e., instruments simultaneously recording about hundreds or thousands of bands across the electromagnetic spectrum) are even more efficient in collecting data at small spatial scales and offer more flexibility in building the training model relating remote sensing data and biotic patterns (Herkül, Kotta, Kutser, & Vahtmäe, 2013; Kotta, Kutser, Teeveer, Vahtmäe, & Pärnoja, 2013). However, the performance of these sensors will be soon limited by the laws of physics. Specifically, the high volume of data generated by an instrument with high spatial and frequency resolution allows only areas in the order of a few thousands km^2^/day to be scanned, even in the case of using experimental high‐speed data links that are not yet commercialized (Villafranca, Corbera, Martín, & Marchán, 2012).

Mapping by remote sensing is based on an assumption that the features of interest in an image reflect or emit light energy in different and often unique ways (Lillesand, Kiefer, & Chipman, 2014) and thereby only the spectrally different features can be mapped. The spectral signatures of submerged benthic vegetation are to a large extent determined by their pigment composition. Although all these macroalgae contain chlorophyll *a*, they have different quantities of other chlorophylls and accessory pigments (Hedley & Mumby, 2002) and thereby are expected to represent unique pigment combinations.

Earlier studies have demonstrated that remote sensing can be successfully applied to predict the occurrence (i.e., presence) of benthic macroalgae, especially if broad taxonomic groups are considered (Andréfouët et al., 2004; Kotta, Remm, Vahtmäe, Kutser, & Orav‐Kotta, 2014), but it is much more challenging to assess patterns of macroalgal species richness, the total cover of macroalgae or the cover of particular species (see Herkül et al., 2013; Kotta et al., 2013 for communities comprised of macroalgae and invertebrates). The optical signature of a remote sensing instrument integrates information from spatial resolutions of meters to tens of meters and thereby consists of mixed signals of the various degrees of green, brown, and red algae either attached on primary substrate or growing epiphytically on other algae. Moreover, changes in spatial arrangement and densities of benthic macroalgae have a strong effect on the outcome as the seafloor may be covered either with small algal patches or lush benthic vegetation (Andréfouët et al., 2004; Hedley & Mumby, 2003).

To date, the existing spectral libraries often consist of averaged reflectance values and ignore spectral variability among macroalgal individuals and taxa (Beach, Borgeas, & Smith, 2006; Vahtmäe, Kutser, Martin, & Kotta, 2006); however, conditions of low or negligible spectral variability among individuals or taxa are rare in nature. To overcome this shortcoming, here we used a novel approach in which we took advantage of natural variability in the pigment composition of macroalgae and used a machine learning approach (see below) to find optimal Sentinel‐2A‐specific decision criteria that can statistically separate macroalgal taxa and predict their richness and cover. Although the Sentinel MSI sensor was designed primarily for terrestrial applications, it can be very useful for aquatic applications (Dörnhöfer, Göritz, Gege, Pflug, & Oppelt, 2016; Toming et al., 2016). To our knowledge, the Sentinel mission has never been used in the mapping of any aquatic environments but offers promising features to efficiently capture biotic signal with high‐end accuracy and reliability.

Machine learning provides a theoretical framework that moves beyond traditional paradigm boundaries by learning from new data (rather than assuming an appropriate data model) and resolving simultaneously a broad range of functions (rather than oversimplifying situations). As machine learning algorithms incorporate inherently “complex realism,” modeling can be seen here as a sophisticated tool to improve our understanding of the patterns of species distribution and particularly the causes of that variation. Specifically, machine learning algorithms have a potential to translate the complex optical signature of a remote sensing instrument into abiotic and biotic features in an ecosystem and thereby reveal identity and patterns of species in remote and largely unexplored regions. Among the novel predictive modeling techniques, boosted regression trees (BRTs) combine the strengths of machine learning and statistical modeling. BRT first relates a response to their predictors by recursive binary splits (regression trees algorithm) and then adaptively combines many simple models to give improved predictive performance (boosting algorithm). Ultimately, the final BRT model can be understood as an additive regression model in which individual terms are simple trees, fitted in a forward, stagewise fashion. As the method avoids overfitting the data, the BRT models are expected to provide robust estimates (Elith, Leathwick, & Hastie, 2008; Hastie, Tibshirani, & Friedman, 2009).

**Figure 1:**
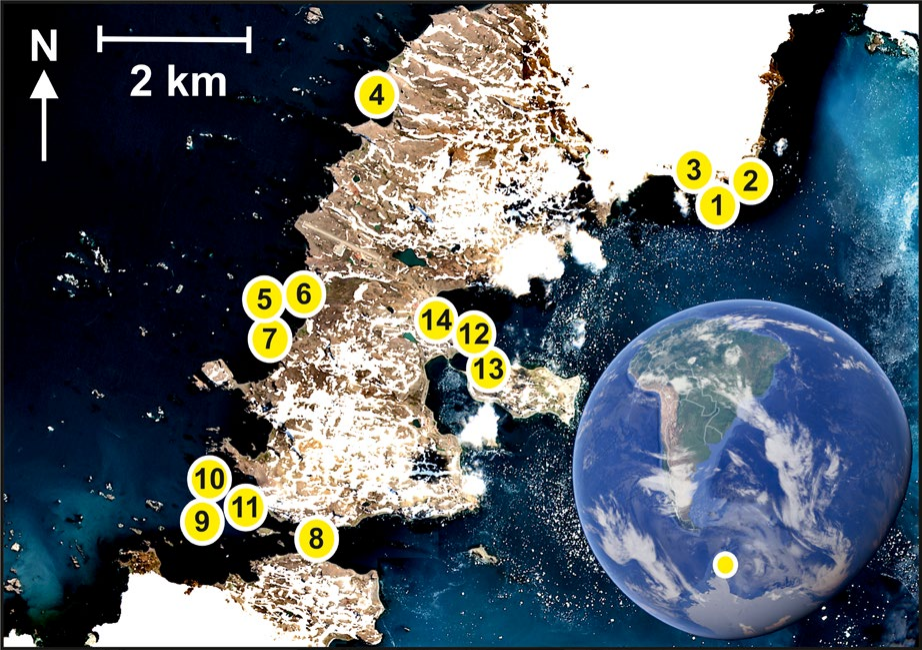
Study area in King George Island, western Antarctic Peninsula. Dots denote the locations of the sampling sites. The background shows a processed reflectance image of the ESA satellite Sentinel‐2A that has been processed using a freeware SNAP version 5.0.0 downloadable at http://step.esa.int/main/download/ [Colour figure can be viewed at http://wileyonlinelibrary.com]

Here, we tested the ecological relevance of the Sentinel‐2A sensor to describe the patterns of intertidal macroalgae of King George Island, Antarctic Peninsula (Figure 1). In order to do so, we quantified *in situ* the cover and richness of the key macroalgal taxa along the full range of intertidal habitats, and then, we used the BRT technique to relate these data to the spectral signal obtained from the sampled locations. For our analyses, we had the following expectations: (a) The Sentinel‐2A sensor captures the signal of the patterns of intertidal macroalgae; (b) although the resolution of the remote sensing instrument is coarser compared to the size of macroalgae and the macroalgal individuals cannot be directly sensed, specific habitat features predict the species richness of intertidal macroalgal communities; and (c) the cover of green, brown, and red algae is indicated by the intensities of reflectance values at specific wavelengths.

## 2 MATERIALS AND METHODS

### 2.1 Macroalgal data

The spatial pattern of intertidal macroalgae was mapped in Fildes Peninsula, King George Island, during the austral summer (January) 2013 (Figure 1; published in Valdivia et al., 2014). Intertidal sessile assemblages are mostly characterized by the red alga *Iridaea cordata*, the brown alga *Adenocystis utricularis*, and the green alga *Urospora penicilliformis*. The sampling followed a nested sampling design in which 10 shores were randomly selected and within each shore, three sites, separated by a few 100 s of meters, were randomly located. Within each site, two patches of substratum separated by 10 s of meters were randomly located, and within each patch, the low, mid, and high intertidal zones were defined. Then, within each combination of patch and tidal zone, three 50 × 50 cm replicate quadrats were randomly located. Within each quadrat, the percentage cover of each macroalgal species was quantified. Taxon richness was calculated as the total number of macroalgal species identified in each quadrat. The sampling plots covered different microhabitats, including emergent rocks, boulders, and shallow tide pools.

### 2.2 Remote sensing data

Sentinel‐2A Level‐1C (L1C) Top of Atmosphere (TOA) data were downloaded from Sentinels Scientific Data Hub (https://scihub.copernicus.eu/; Table 1). Sentinel‐2A cloud‐free images with 20 m resolution were used for analyses. Sentinel‐2A Toolbox (S2TBX) version 5.0.1 in Sentinel Application Platform (SNAP) version 5.0 on Windows 10 (64 bit) was used to process the images. 1 × 1 and 3 × 3 cloud‐free pixels were extracted from each sampling point. The Sen2Cor 2.5.5 atmospheric correction module was applied to convert TOA into the bottom of atmosphere (BOA) reflectance.

**Table 1:** Spectral bands, central wavelengths (nm), bandwidths (nm), and appropriate spatial resolutions (m) of Sentinel‐2A MSI sensor

Band name	Central wavelength	Bandwidth	Spatial resolution
B1	443	20	60
B2	490	65	10
B3	560	35	10
B4	665	30	10
B5	705	15	20
B6	740	15	20
B7	783	20	20
B8	842	115	10
B8a	865	20	20
B9	945	20	60
B10	1375	30	60
B11	1610	90	20
B12	2190	180	20

### 2.3 Analyses

Macroalgal and remote sensing data were joined based on geographical proximity. When linking ground truth and remote sensing data, we used the archived snapshot of the reflectance data that were temporally closest to the ground truth data. A sampling in the same day is usually not possible due to high number of cloudy days in Antarctica. Nevertheless, the lack of exact timing is not an important source of error as within‐season and even between‐year variability of intertidal macroalgal patterns is not large in Antarctica. An average of 18 quadrats (three replicate quadrats at each combination of patch and tidal zone) was used to characterize one sentinel pixel. The relationships between different remote sensing variables (i.e., the BOA reflectance of different remote sensing bands shown in Table 1), richness, and percentage cover of intertidal green, brown, and red macroalgae were explored using the BRT technique. The BRT is a technique that combines the strength of machine learning and statistical modeling; it avoids starting with a data model and rather uses an algorithm to learn the relationship between the response and its predictors (Hastie et al., 2009). The predictive performance of BRT models is superior to most traditional modeling methods. The BRT iteratively develops a large ensemble of small regression trees constructed from random subsets of the data. Each successive tree predicts the residuals from the previous tree to gradually boost the predictive performance of the overall model. The final BRT model is a linear combination of many trees (usually hundreds to thousands) that can be thought of as a regression model where each term is a tree. Although BRT models are complex, they can be summarized in ways that give powerful ecological insight (Elith et al., 2008; Kotta et al., 2017). In fitting a BRT, the learning rate and the tree complexity must be specified. The optimum model was selected based on model performance, with learning rates, number of trees, and interaction depth set at 0.001, 3000, and 5, respectively. In order to avoid potential problems of overfitting, unimportant variables were dropped using a simplify tool. Such simplification is most useful for small datasets where redundant predictors may degrade performance by increasing variance. Model performance was evaluated using the cross‐validation statistics calculated during model fitting (Hastie et al., 2009).

In order to assess the transferability of the developed models, we used another macroalgal dataset collected from 13 sites during late January and early February 2017 to demonstrate how well the developed models predicted macroalgal cover and richness when applied outside of the training data. The collection of remote sensing data and linking of the BOA reflectance values to the ground truth data were performed as described above. The quality of the models was assessed using a simple linear regression fitting.

We expected that wetting and drying cycles have only marginal effects in our models as green algae are essentially uncovered with water, whereas other algal groups are mostly covered with water. Thus, within macroalgal groups, we do not expect large differences in wetting. Moreover, the environmental conditions in the Antarctic Peninsula are cold and humid. Thus, even during low tide, algae always remain wet due to the almost complete lack of solar energy. Similarly, the BRDF effects are expected to be minor as virtually all intertidal algae are very small. Ultimately, as we used a statistical approach that quantified typical reflectances of the studied macroalgal groups, the wavelengths that potentially incorporated the above effects would have been excluded from the final models due to high noise‐to‐signal ratio.

## 3 RESULTS

**Figure 2:**
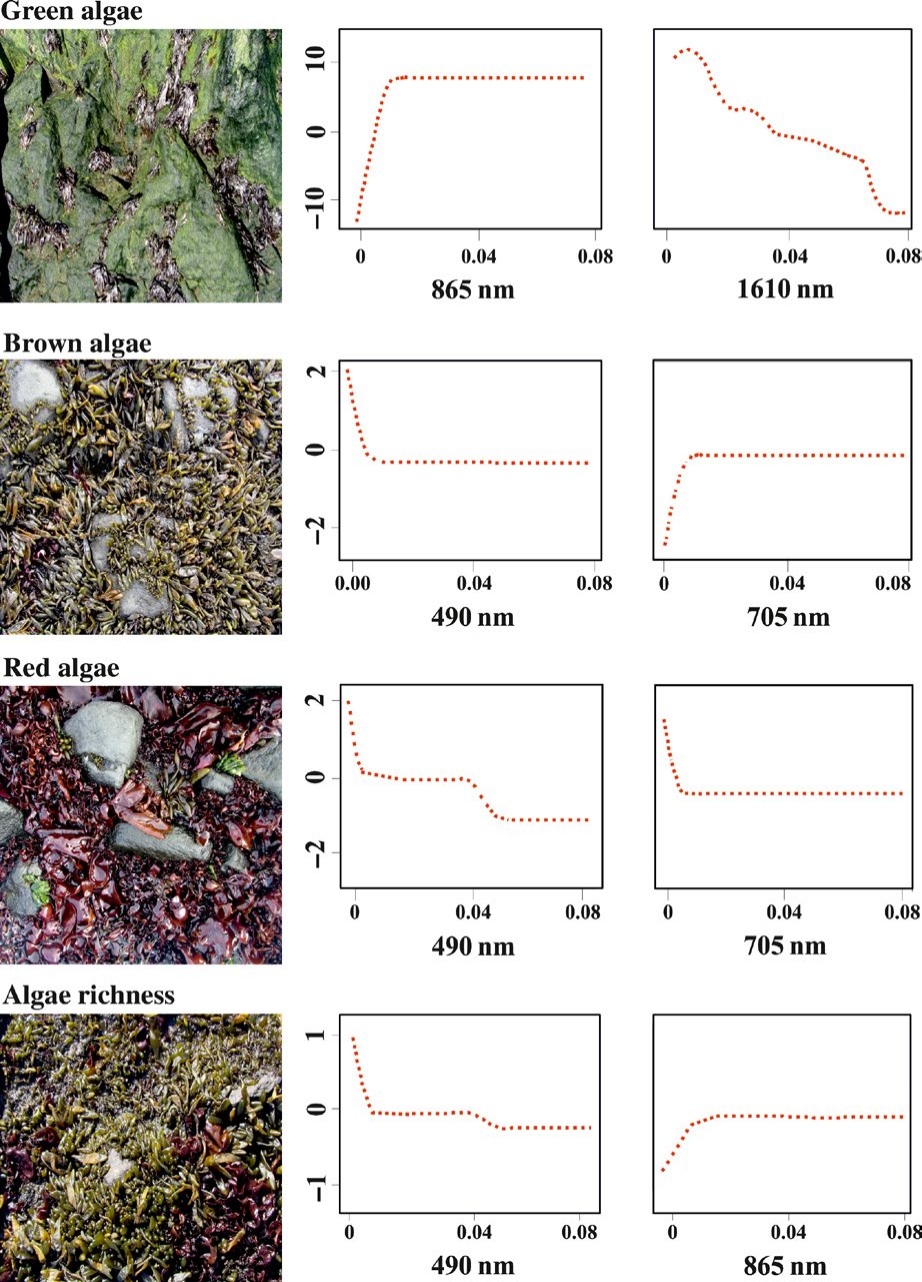
Left: Photographs of the key intertidal benthic taxa in the study area. Right: Partial dependence plots for the two most influential remote sensing variables (*x*‐axis: the bottom of atmosphere reflectance at an indicative wavelength) in the model for the cover or richness of different macroalgal taxonomic groups (*y*‐axis: marginal effect on logit (p)) [Colour figure can be viewed at http://wileyonlinelibrary.com]

Our BRT models on a pooled dataset showed that the Sentinel‐based remote sensing described a significant proportion of variability in the cover and richness of Antarctic macroalgae (Figure 2; Table 2). The highest performing models were for macroalgal richness (*r* ^2^ = 0.49) and the cover of green algae (*r* ^2^ = 0.45) as opposed to the model of brown (*r* ^2^ = 0.40) and red algal cover (*r* ^2^ = 0.31).

**Table 2:** The percentage of total variance explained by the BRT models (in bold) and the relative contribution of different remote sensing bands to total variance (summing up to 100%)

Model and model variables	% variability explained
**Green algae model**	**45**
BOA1 × 1_20m_865	26.5
BOA1 × 1_20m_1610	25.0
BOA1 × 1_20m_490	17.4
BOA1 × 1_20m_560	17.4
BOA1 × 1_20m_665	8.6
BOA1 × 1_20m_705	4.4
BOA1 × 1_20m_740	2.6
**Brown algae model**	**40**
BOA1 × 1_20m_490	49.7
BOA1 × 1_20m_705	28.8
BOA1 × 1_20m_560	21.5
**Red algae model**	**31**
BOA1 × 1_20m_490	36.9
BOA1 × 1_20m_705	18.3
BOA1 × 1_20m_1610	11.8
BOA1 × 1_20m_560	10.7
BOA1 × 1_20m_665	9.6
BOA1 × 1_20m_783	4.8
BOA1 × 1_20m_865	4.6
BOA1 × 1_20m_740	3.32
**Species richness model**	**49**
BOA1 × 1_20m_490	41.4
BOA1 × 1_20m_865	23.6
BOA1 × 1_20m_1610	19.2
BOA1 × 1_20m_560	15.8

Macroalgal richness was best predicted by reflectance values at 490 and 865 nm. If higher reflectances at 490 nm predicted lower macroalgal richness, then an inverse relationship was found at 865 nm.

The intensity of reflectance at 865 nm increased logistically with the elevated cover of green algae. At a higher wavelength of 1,610 nm, however, the relationship between reflectance and green algal cover was opposite. Brown and red algae were identified at 490 nm, and higher reflectance values at this wavelength predicted lower algal biomasses. Besides, the brown and red algae can be identified by characteristic features in their reflectance at the red end of the spectra (around 700 nm). The latter is also true for green algae but as their reflectance values were very high in the infrared spectrum of light, the red area of the spectrum had a marginal indicative value.

**Figure 3:**
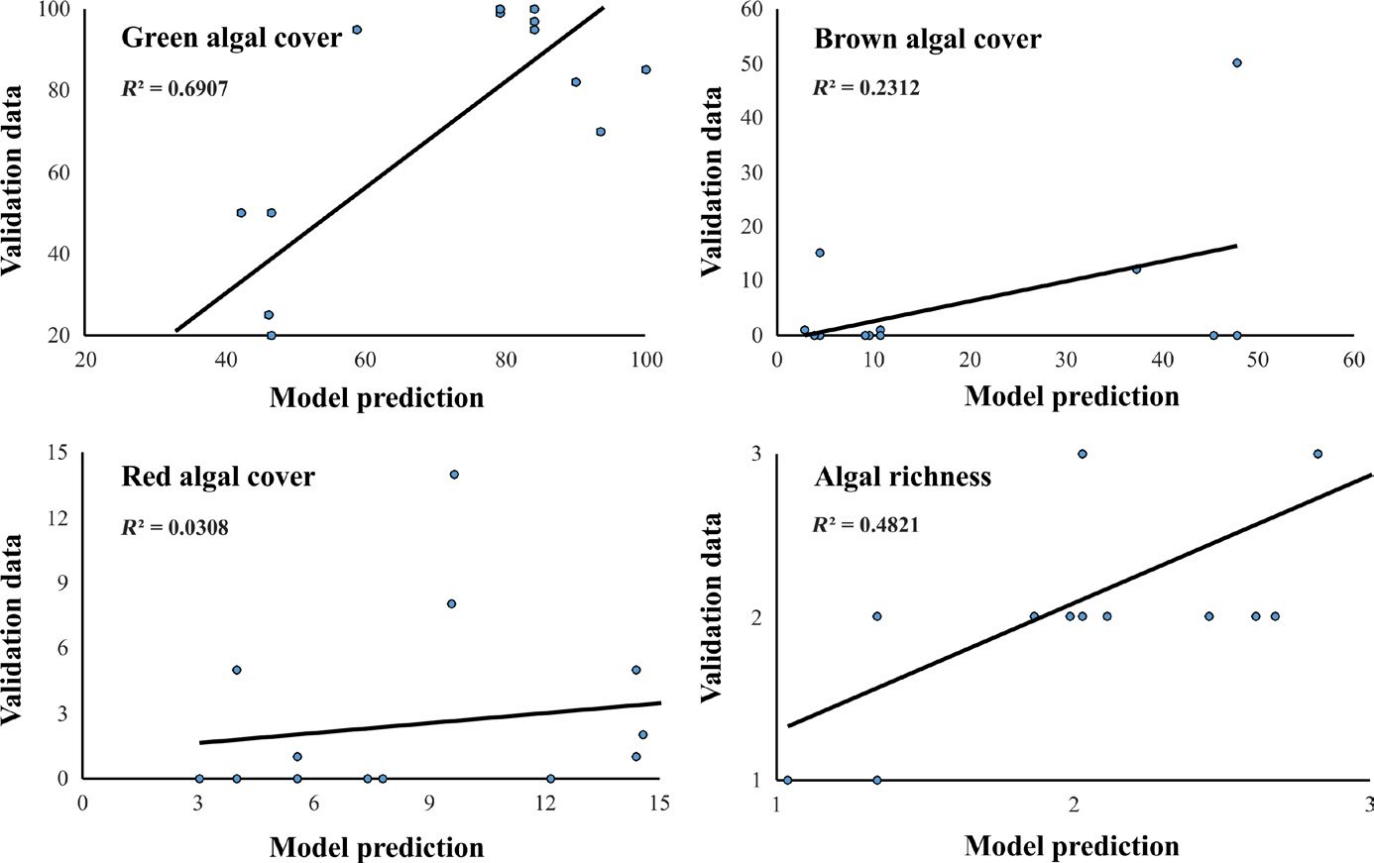
Interpolation test for assessing the performance of the developed models at different sites for which ground truth data were also available. *X*‐axis: percent coverage of different macroalgal taxonomic group or macroalgal richness estimated by BRT modeling, and *y*‐axis: percent coverage of different macroalgal taxonomic group or macroalgal richness estimated during a separate field survey; *R* ^2^: the coefficient of determination of linear regression fitting [Colour figure can be viewed at http://wileyonlinelibrary.com]

When applying the developed models at different sites for which ground truth data were also available, models that explained better training data were also more powerful in predicting macroalgal spatial patterns under novel conditions. The models of green algae and macroalgal richness predicted a similar percentage of total variability of both training and validation data. The other two models, however, performed poorly under novel conditions (Figure 3).

## 4 DISCUSSION

In the current study, we demonstrate a methodology that allows a statistically significant separation of spectral signatures of benthic intertidal macroalgae. Our analyses also showed that the shapes of functional‐form relationships of reflectance spectra at specific wavelengths with macroalgal species richness, and with cover of green, brown, and red algae, were consistent.

Distinctive spectral signatures of the studied macroalgae could often be inferred from the knowledge of the characteristic pigments present in different macroalgal groups. Although the morphology, thickness of thalli, and cellular architecture may affect the relationship between pigment densities, absorption, and thus reflectance spectra (Hannach, 1989; Ramus, 1978; Vogelmann & Björn, 1986), such natural variability did not hinder us from demonstrating some clear‐cut differences in spectral signatures between different taxonomic groups of macroalgae. The photosynthetic pigments of green algae are chlorophylls *a* and *b* that absorb strongly in the blue and red part of the spectrum, and thereby produce low reflectance values in the bands from 400 to 500 nm as well as from 650 to 680 nm (Haxo & Blinks, 1950). Our study showed a distinct reflectance pattern at these wavelengths, but as the reflectance values of green algae were very high in the infrared spectrum of light, the blue and red area of the spectrum had only a marginal indicative value. The cover of green algae was best predicted by reflectances at the near‐infrared spectrum of light. The established functional‐form relationships largely varied among the studied wavelengths. Lower wavelengths (865 nm) were indicative of green algae and thereby increased reflectance intensities at these wavelengths corresponded to the elevated cover values of green algae. At higher wavelengths (1,610 nm), bare substrates had higher reflectance compared to green algae, and therefore, the relationship was opposite at this part of the spectrum.

Brown and red algae were best predicted at 490 nm. The basic mechanism behind this relationship is that the primary pigment in plants (i.e., chlorophyll) absorbs light the most in the blue regions of the visible light spectrum, and therefore, areas with higher plant cover are characterized by lower reflectances at this spectral range (e.g., Anderson & Barrett, 1979; Haxo & Blinks, 1950). Brown and red algae are known to have clear reflectance peaks between 600 and 650 nm (Kotta et al., 2014). However, the Sentinel‐A satellite does not have appropriate detection wavelength bands to separate such reflectance peaks. This suggests that hyperspectral instruments should be used instead to ensure appropriate detection of the pigments of brown and red algae (e.g., Vahtmäe & Kutser, 2013). Moreover, red light is absorbed more strongly by water compared to shorter wavelength light. Consequently, the absolute reflectance values of brown and red algae are considerably lower than that of green algae making the detection of these algal groups more challenging.

The remote sensing signal of macroalgal richness is expressed as a sum of the multitude of optically differing species that are confined at the same location. Due to coarser spatial resolution of the satellite sensors compared to the size of macroalgae, the patchiness of the reflectance spectra of remotely sensed images may only hint at algal richness (Herkül et al., 2013). Nevertheless, the total cover of green and brown algae seems to be a good proxy of macroalgal richness in the Antarctic region as shown by a strong linkage between macroalgal richness and reflectances at 490 and 865 nm.

Our study also showed that the predictive performance of statistical models varied among green, brown, and red algae. This difference in predictive performance is due to better detection of objects that are situated on the upper shore and/or at the top of biota (such as green algae). As green algae are located high in the intertidal zone and often covered only by a thin layer of seawater, these algae are easily identified in the region of the infrared spectrum of light (Kutser, Dekker, & Skirving, 2003; Kutser, Vahtmäe, & Martin, 2006). The Sentinel‐2 MSI sensor has spectral bands in the red edge and near‐infrared parts of spectrum and, as shown above, this gives the MSI sensor a significant advantage over other satellite sensors in detecting and classifying slightly submerged and emergent benthic vegetation. The models of brown and red algae performed poorly, and this stems from generic low reflectance of brown and red algae as well as the lack of appropriate detection wavelength bands of the Sentinel‐A satellite. The latter is a significant limitation of the current method and can be alleviated by a wider usage of hyperspectral space missions.

Although our statistical models reproduced many of the observed biotic patterns, a large part of the total variability of the observed macroalgal data remained unexplained. Currently, the remote sensing instruments operate at the spatial scale of 10 s of meters, whereas important components of variability of biotic patterns occur at the scale of individual meters. Thus, in order to achieve a full potential of models developed in this manuscript, the usage of better satellite images at higher resolution is also advised.

This single‐case study indicates many limitations concerning the generalizations that can be made from these results. Nevertheless, we clearly demonstrated that remote sensing, when combined with *in situ* mapping and machine learning algorithms, provides a powerful tool to identify sites hosting intertidal macroalgae and, what is even more important, to predict the cover and richness of these macroalgae. The models vary greatly in the results but considering the fast development of remote sensing applications (increased number of hyperspectral remote sensing missions operating at fine spatial scales) many of such statistical uncertainties can be removed in the near future.

By pooling all available georeferenced data in the continent, the generality of models can be improved to a large extent, thereby giving us the opportunity to map all intertidal macroalgal habitats and monitor their dynamics during all vegetative seasons in Antarctica. This is a significant milestone as many coastal areas in Antarctica are difficult to access and most scientific visits occur in summer months only. We still have to assess the application of this method to other marine habitats—such as shallow‐subtidal areas—for which baseline data and evidences of climate change‐related dynamics are available (Quartino, Deregibus, Campana, Latorre, & Momo, 2013; Mystikou et al., 2014; Campana et al. 2018). When continuous fine‐resolution large‐scale observations become available, a series of novel hypotheses can be formulated and tested, which can be considered as directions for future research with important potential. To forecast responses to global climate change, we need to learn (a) how rates of species range expansion and extinctions compare among a wide range of regions, (b) which macroalgal habitats respond first, and (c) whether there are irreversible environmental thresholds where the return to the original state is no longer likely.

## COMPETING INTERESTS

The authors declare no competing interests.

## AUTHORS’ CONTRIBUTIONS

JK and NV conceived the study; JK and NV obtained funding; NV, TK, and KT collected data; JK, NV, MR, TK, KT, and HOK analyzed the data; JK and NV led the writing of the manuscript; and all authors contributed critically to the drafts and gave final approval for publication.

## DATA AVAILABILITY

The datasets that were generated and/or analyzed during the current study are freely available from the corresponding author upon request and are archived in the Dryad Data Repository: https://doi.org/10.5061/dryad.33cd137.
